# Coevolutionary analyses of the relationships between piroplasmids and their hard tick hosts

**DOI:** 10.1002/ece3.685

**Published:** 2013-07-30

**Authors:** Huitian Gou, Guiquan Guan, Aihong Liu, Miling Ma, Ze Chen, Zhijie Liu, Qiaoyun Ren, Youquan Li, Jifei Yang, Hong Yin, Jianxun Luo

**Affiliations:** State Key Laboratory of Veterinary Etiological Biology, Key Laboratory of Veterinary Parasitology of Gansu Province, Key Laboratory of Grazing Animal Diseases MOA, Lanzhou Veterinary Research Institute of Chinese Academy of Agricultural ScienceXujiaping 1, Lanzhou, Gansu, 730046, China

**Keywords:** Coevolution, COI, hard tick, piroplasmid

## Abstract

Host–parasite coevolution is a key driver of biological diversity. To examine the evolutionary relationships between piroplasmids and their hard tick hosts, we calculated the molecular clock and conducted phylogenetic analyses of both groups. Based on our results, we conclude that the divergence time of piroplasmids (∼56 Mya) is later than divergence time of their hard tick hosts (∼86 Mya). From analyses of the evolution of both piroplasmid and vector lineages and their association, we know that hard ticks transmit piroplasmids with high genus specificity and low species specificity.

## Introduction

**Figure 1:**
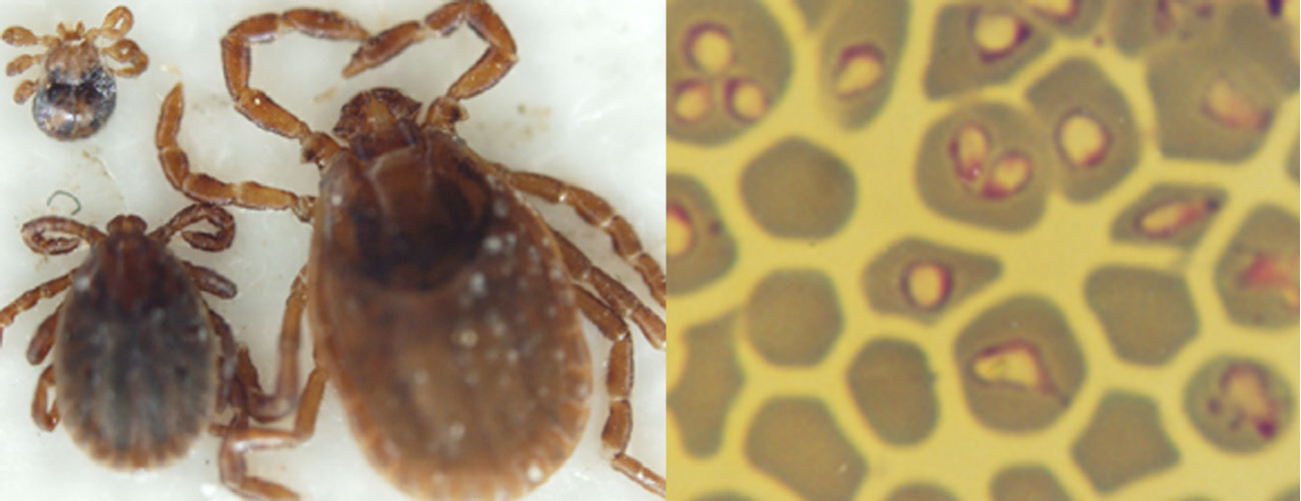
Piroplasmids and their hard tick hosts.

Coevolution between parasites and their hosts is a widely recognized phenomenon in many host–parasite systems. It is also an essential component of evolution and the diversity of life. Piroplasmids (*Babesia* spp. and *Theileria* spp.) are protozoan hemoparasites of great economic, veterinary, and medical importance worldwide. They infect a diverse array of mammalian and a few avian hosts (Lack et al. 2012; Schnittger et al. 2012). Their definitive hosts, species of hard ticks, are obligate ectoparasites, and seem to be second in importance only to mosquitoes as vectors of human and animal diseases (Fig. 1) (Fivaz et al. 1992; Jongejan and Uilenberg 2004). Hard ticks are exclusively hematophagous and are distributed worldwide. Each piroplasmid species is transmitted by different hard tick species, even those piroplasmids with close phylogenetic relationships (Yin et al. 2002).

The diversity of the relationships between host and parasite population structures (such as host switching, cospeciation, duplication, and extinction) usually makes it difficult to draw general conclusions and underlines the need for more studies on host–parasite systems (Garamszegi 2009; Ricklefs et al. 2004). Therefore, systematic studies of the diversification of piroplasmids in relation to their vector can provide insight into the coevolutionary events that shaped present-day interactions among these taxa.(Agudo et al. 2010).

Previous studies estimated that the date of origin of the Apicomplexa was between 1100 and 500 Mya based on molecular clock estimations (Berney and Pawlowski 2006; Escalante and Ayala 1995), predating by far the presumed origin of the ticks as well as the radiation of mammals and birds (120–90 Mya) (Escalante and Ayala 1995). Given that apicomplexan phylogeny correlates with the taxonomy of their definitive host, hard ticks probably represent the primordial host of piroplasmids (Barta 1989). Thus, the question arises as to when an ancient piroplasmid ancestor might have selected the hard tick as its vector. MtDNA offers several advantages in comparison to nuclear DNA: rapid evolution, limited exposure to recombination, lack of introns, and high copy number. These characteristics of mtDNA are important for routine amplification by polymerase chain reaction (PCR) and use as a molecular marker for phylogeny (Gou et al. 2012). In this study, we used the cytochrome oxidase I (*COI*) gene to calculate the molecular clock of piroplasmids and hard ticks, and also reconstructed molecular phylogenies for the parasites and their hosts to assess the extent of coevolution in this association.

## Materials and Methods

### Taxa sampling

In total, 11 piroplasmid species were collected from five provinces and three autonomous regions (Gansu, Henan, Yunnan, Hebei, Sichuan, Xinjiang, Ningxia, and Inner Mongolia) of China. Cattle and sheep were inoculated with 10-mL cryopreserved infectious blood containing each isolate. When the level of parasitemia reached more than 5%, blood from the jugular vein was collected into tubes using heparin as an anticoagulant. Parasites were isolated as described in previous studies, and stored at −70°C for DNA extraction.

Correspondingly, 10 species of hard tick were also collected from field sites and different hosts in various regions (Gansu, Xinjiang, Xizang, Guangxi, Qinghai, Hubei, and Henan) of China. After morphological identification, these ticks were stored in 100% ethanol and conserved at 4°C. To eliminate interference from the specimens, only female and unfed adult hard ticks were used in the study.

### DNA extraction, PCR amplification, and DNA sequencing

Parasite DNA was isolated using a QIAamp DNA MiniKit (Qiagen, Hilden, Germany), according to the manufacturer's instructions. The fragments of *COI* gene (935–1458 bp) were amplified from the piroplasmid using PCR and the following four pairs of primer, Bafor1: 5′-ATAGGATTCTATATG-AGTAT-3′; Barev1: 5′-ATAATCAGGTATTCTCCTTGG-3′; Bafor2: 5′-TCTCTTCATGGTTTAAT-TATGATAT-3′; and Barev2: 5′-TAGCTCCAATTGATAAAACA-AAGTG-3′, were designed according to the sequence of *COI* gene of *Babesia bovis* (AB499088) and *Babesia bigemina* (AB499085). And Thfor1: 5′-GGAAATCATAAAATTATTGGTATA-3′, Threv1:5′-CATCAGGATAATCTGGTATTCTTCT-3′, Thfor2: 5′-TGGCTTGCTT-ATTGGTTTGGT-3′, and Threv 2:5′-C-AACATCATAGTAGCTCCAA-3′) were designed according to the sequence of *COI* gene of *Theileria orientalis* (AB499090) and *T. parva* (AB499089). In addition, the sheep and cow genomes were used to check the specificity of the primers.

DNA of hard ticks was extracted using a tissue kit (Qiagen). Each sample was cut with sterile scissors within a 1.5 mL microtube. After digestion with proteinase *K* (20 mg/mL), the samples were applied to the columns for DNA absorption and washing. Finally, the DNA was eluted in 100 mL of elution buffer provided in the kit and stored at −20°C. The universal primers used for PCR were as follows: LCO1490: 5′-GGT-CAACAAACATAAAGATATTGG-3′ and HCO2198: 5′-TAAACTTCAGGGTGACCAAAAAATCA-3′(Vrijenhoek 1994).

PCR reactions were run using the following thermal cycling program: 94°C for 5 min, 35 cycles at 94°C for 1 min, 45–54°C for 1 min, and 72°C for 1 min, followed by a final extension at 72°C for 8 min. The 50-μL PCR reaction mixture included 37.5 μL of ultrapure water, 5 μL of 5× PCR buffer, 1 μL of each primer (20 μmol/L), 4 μL of dNTPs (10 mmol/L), 0.5 μL of Taq polymerase (5 U), and 1 μL of the DNA template. PCR products were purified using a PCR purification kit (Takara Bio Inc, Japan). Sequencing reactions were resolved on an ABI 3730 automated DNA sequencer.

Other *COI* gene sequences of piroplasmids, hard ticks, and relevant outgroup (*Plasmodium* spp. and *Ornithodoros* spp.) were obtained from GenBank (Table S1).

### Sequence alignment

Edited sequence contigs for both *COI* genes (from piroplasmids and from hard ticks) were aligned using the software program MEGA 4.0 (Tamura et al. 2007) with default settings. To avoid bias in refining alignments, ambiguous alignment positions, including the beginning and end regions, were excluded. Furthermore, nucleotide sequences were translated into amino acid sequences using the invertebrate mitochondrial code in MEGA 4.0 to prove the correctness of the DNA sequences.

#### Saturation test

Given that saturation in substitutions can lead to incorrect phylogenetic inferences, the *COI* genes from piroplasmids and hard ticks were evaluated for transition and transversion substitutions by DAMBE 5.3.3(Xia and Xie 2001).

### Calibration point

Owing to a lack of fossil piroplasmids and potentially many unsampled lineages, we generated a combined alignment of piroplasmid and *Plasmodium COI* sequences and calibrated the phylogeny with an age of 12.82 million years (95% highest posterior density [HPD] 9.93–19.49 Mya) for the three most recent common ancestors (MRCA) of *Plasmodium* spp., as reported previously (Ricklefs and Outlaw 2010).

For hard ticks, three fossils were used as calibration points: the MRCA of the clade including *Ixodes pavlovskyi*, *Ixodes persulcatus*, *Ixodes bakeri*, *Ixodes cornuatus*, *Ixodes ricinus*, and *Ixodes hirsti* based on amber *Ixodes succineus* (35–40 Mya) (Weidner 1964); the MRCA of the clade including *Hyalomma truncatum*, *Hyalomma marginatum*, *Hyalomma rufipes*, *Hyalomma lusitanicum*, *Hyalomma dromedarii*, *Hyalomma asiaticum asiaticum*, and *Hyalomma asiaticum* based on amber *Hyalomma* sp. (35–50 Mya) (De La Fuente 2003); and the MRCA of the clade including *Amblyomma triguttatum*, *Amblyomma pattoni*, and *Amblyomma variegatum* based on amber *Amblyomma* sp. (15–40 Mya) (Poinar 1992).

### Divergence time and phylogenetic analyses

ModelTest 3.7 (Posada 2005) was used to determine the appropriate DNA substitution model and gamma rate heterogeneity with the akaike information criterion (AIC). For both matrices, ModelTest was performed for each gene region. As a result, the GTR + I + G was determined as the best-fitting statistical model.

BEAST 1.7.1 (Drummond and Rambaut 2007) was used to conduct relaxed-clock Bayesian analyses to estimate topology and divergence times simultaneously. Evolutionary rates along branches followed an uncorrelated lognormal distribution, and a Yule speciation process was imposed for all analyses. Two independent MCMC (Markov chain Monte Carlo) runs were performed, of 10 million and 5 million generations, with sampling every 1000 generations. These two separate runs were then combined (following the removal of 10% burn-in) using LogCombiner 1.7.1. Adequate sampling and convergence of the chain to stationary distribution were confirmed by inspection of MCMC samples using Tracer 1.5 (Drummond and Rambaut 2007). The effective sample size (ESS) values of all parameters were greater than 200, which were considered a sufficient level of sampling. The sampled posterior trees were summarized using TreeAnnotator 1.7.1 to generate a maximum clade credibility tree (maximum posterior probabilities [PP]) and calculate the mean ages, 95% HPD intervals, PP, and substitution rates for each node. The BEAST topology was visualized with FigTree 1.3.1 (Drummond and Rambaut 2007).

## Results

### Data characteristics

We collected 14 species of piroplasmid (eight species of *Babesia* and six species of *Theileria*). Nucleotide sequence editing and alignment resulted in *COI* data partitioning of 950 characters. In the aligned sequences, 466 sites were variable and 388 sites were parsimony informative. First-, second-, and third-codon positions showed AT biases of 58.6, 60.6, and 84.2%, respectively. Codon usage frequency was 33.3%.

In total, 31 species of hard tick were used in this study (six species of *Ixodes*, five species of *Haemaphysalis*, three species of *Dermacentor*, seven species of *Hyalomma*, three species of *Amblyomma*, and seven species of *Rhipicephalus*). All sequences were aligned with a consensus length of 586 bp, 303 sites were variable and 260 sites were parsimony informative. First-, second-, and third-codon positions showed AT biases of 57.5, 57.5, and 85.9%, respectively. Codon usage frequency was 32.3%.

### Saturation effects and substitution patterns

**Figure 2:**
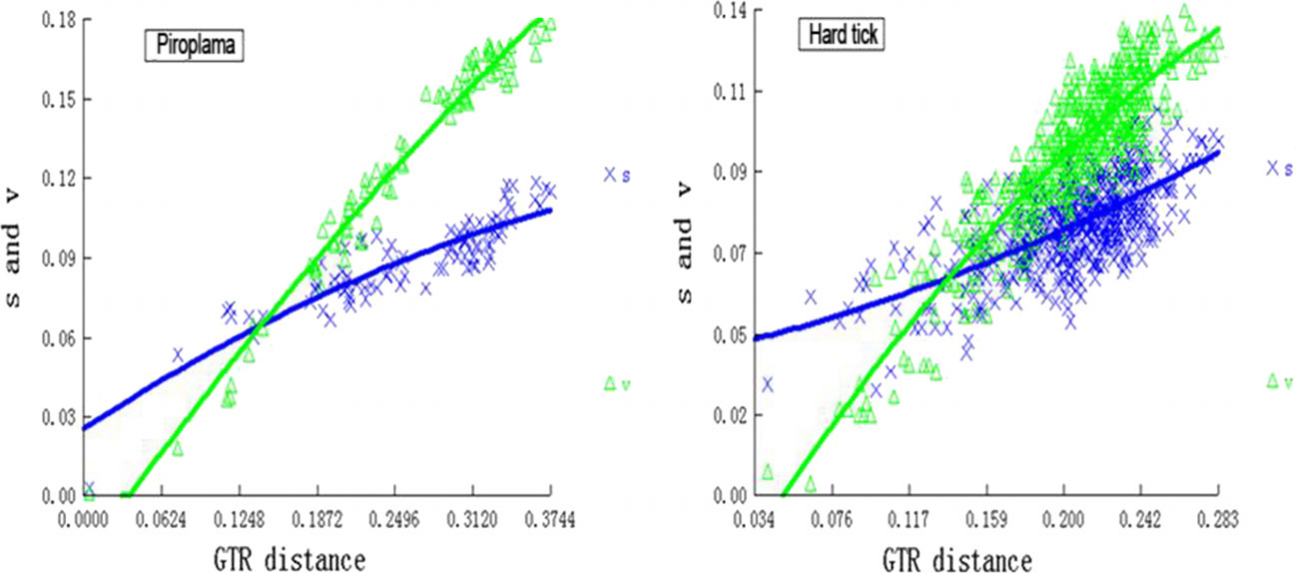
Substitution patterns of the cytochrome oxidase I (COI) gene. The number of transition (S) and transversion (V) substitutions is plotted against the Kimura-2 parameter (K-2P) distance considering all sites. Each point represents a pairwise comparison between two taxa.

Comparison of the transition and transversion frequencies of piroplasmids and hard ticks revealed that the frequency of transition was lower than transversion (Fig. 2). For the test of substitution saturation, which examined whether the observed Iss (index for saturation substitution) was significantly less than Iss.c (critical Iss), we found that the observed Iss (0.58) was less than the Iss.c (0.76) for piroplasmids, and Iss (0.27) was less than the Iss.c (0.74) for hard tick. These results indicated that the two *COI* genes were appropriate for phylogenetic reconstruction.

### Estimating divergence time

**Figure 3:**
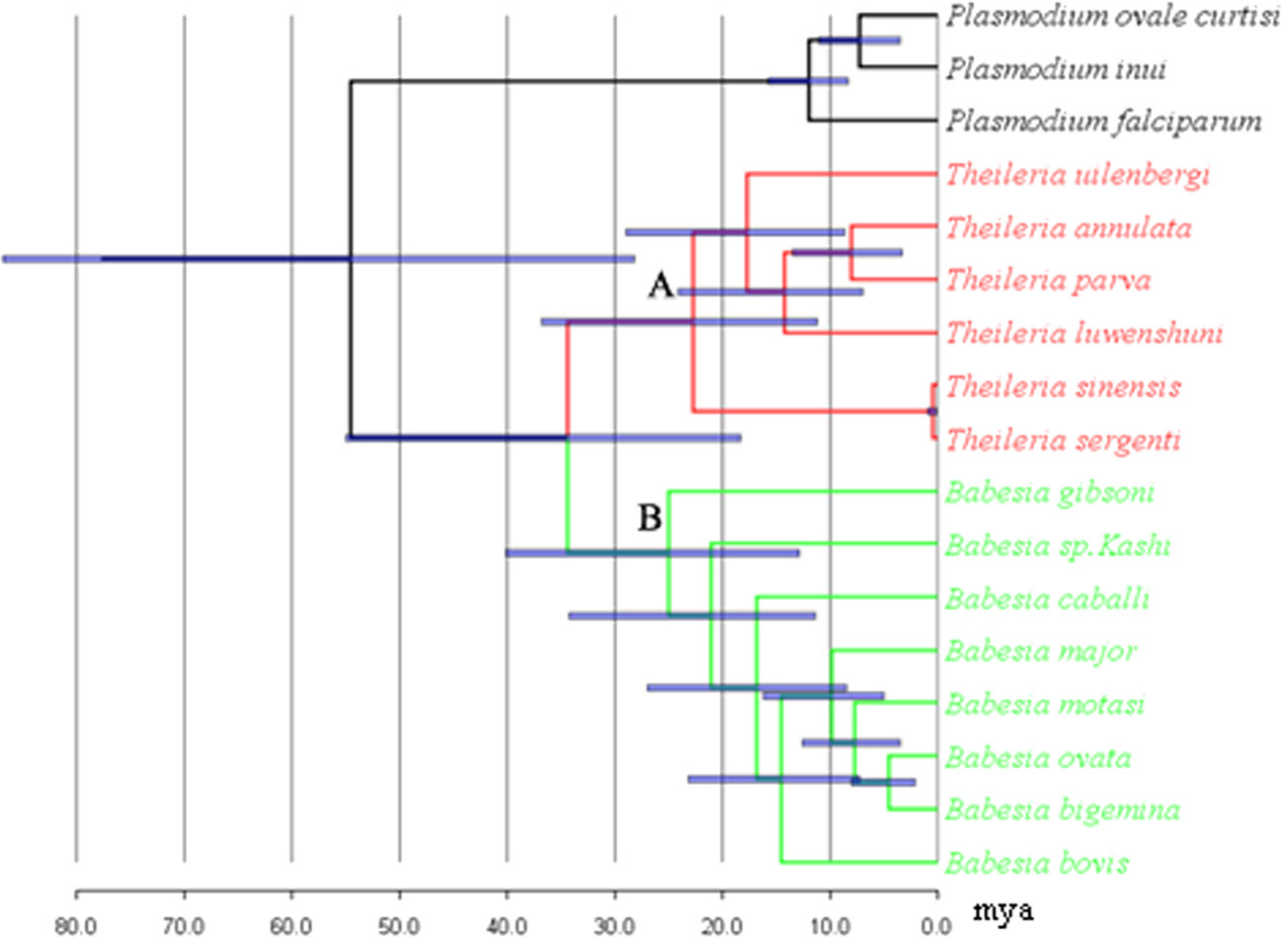
Chronogram resulting from the molecular dating analysis of piroplasmid conducted in BEAST 1.7.1. Shaded bars represent the 95% highest posterior density interval for divergence estimates.

The analyses estimated the divergence time of piroplasmids to be during the Paleocene, ∼56.48 Mya (95% HPD 28.17–86.87); node A represented *Theileria* with a node age of 23.38 Mya (95% HPD 11.11–36.71) and node B represented *Babesia* with a node age of 25.74 Mya (95% HPD 12.75–40.73) (Fig. 3, Table 1). The *Babesi* clade formed slightly earlier than that of *Theileria,* and the two events both began during the late Eocene.

**Table 1:** Posterior age distributions of the major nodes of piroplasmids and hard ticks using BEAST analyses

Node	Genus	Mean (Mya)	95% highest posterior density (Mya)
A	*Theileria*	23.38	11.11–36.71
B	*Babesia*	25.74	12.75–40.73
C	*Ixodes*	46.71	39.95–53.45
D	*Haemaphysalis*	46.58	35.29–58.60
E	*Dermacentor*	35.72	26.50–45.38
F	*Hyalomma*	35.42	28.92–41.89
G	*Amblyomma*	34.85	30.03–39.96
H	*Rhipicephalus*	37.72	30.01–45.82

**Figure 4:**
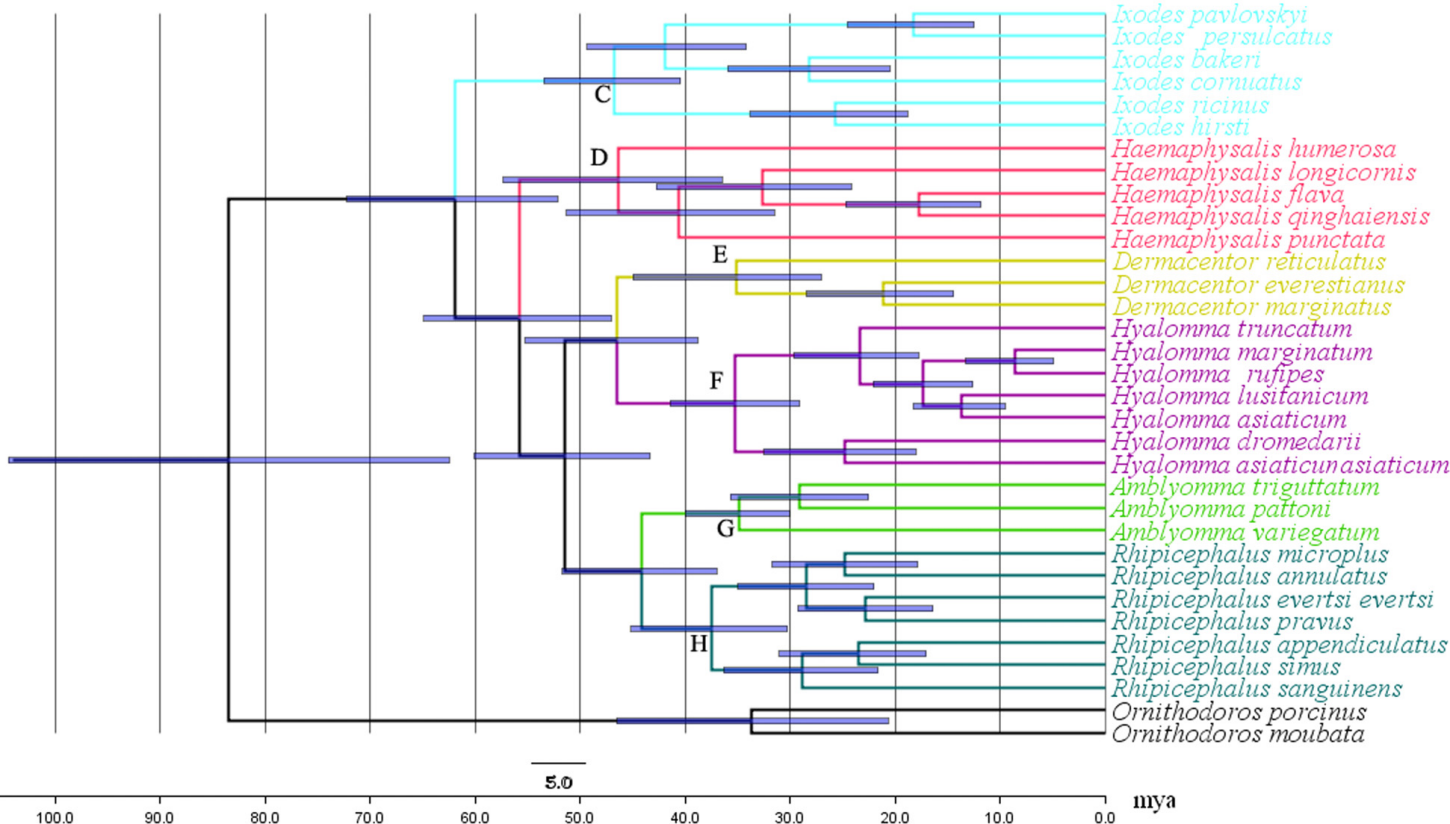
Chronogram resulting from the molecular dating analysis of hard ticks conducted in BEAST 1.7.1. Shaded bars represent the 95% highest posterior density interval for divergence estimates.

The analyses estimated that hard ticks began to diverge during the Cretaceous, ∼86 Mya (95% HPD 61.11–104.16 Mya). The nodes C, D, E, F, G, and H in Figure 4 represent the times at which the following genera formed: *Ixodes* (46.71 Mya; 95% 39.95–53.45), *Haemaphysalis* (46.58 Mya; 95% HPD 35.29–58.60), *Dermacentor* (35.72 Mya; 95% HPD 26.50–45.38), *Hyalomma* (35.42 Mya; 95% HPD 28.92–41.89), *Amblyomma* (34.85 Mya; 95% HPD 30.03–39.96), and *Rhipicephalus* (37.72 Mya; 95% HPD 30.01–45.82). All the genera began to form during the early Eocene–late Paleocene.

### Phylogenetic analyses

The analyses of piroplasmids and hard ticks resulted in a robust phylogeny. For the piroplasmids, the relationships among species of *Babesia* and *Theileria* were well resolved in the Bayesian inference of the nuclear data. Two benign bovine *Theileria* species (*T. sinensis* and *T. sergenti*) fell into one strongly supported clade (PP = 1.00). Meanwhile, another two pathogenic bovine species (*T. annulata* and *T. parva*) were also strongly grouped (PP = 1.00). The relationships among the *Theileria* spp. were as follows: (*T. sinensis* + *T. sergenti*) + (*T. uilenbergi* + (*T. lunwenshuni* + (*T. annulata* + *T. parva*))). For the eight species of *Babesia*, the relationships of these clades were as follows: (*B. gibsoni* + (*Babesia* sp. Kashi + (*B. caballi* + (*B. bovis* + (*B. major* + (*B. motasi* + (*B. ovate* + *B. bigemina*))))))) and nodes were well supported by 1.00, 0.90, 1.00, 0.52, 1.00, 0.97, and 1.00, respectively (Fig. 3). For the hard ticks, six genera formed a monophyletic group. The *Ixodes* genus has distinct morphological differences compared with the other genera of hard tick, and formed a single clade. The relationships of these hard tick clades were as follows: (*Ixodes* + (*Haemaphysalis* + (*Dermacentor* + *Hyalomma*) + (*Amblyomma* + *Rhipicephalus*))) and nodes were well supported by 0.95, 0.93, 0.59, 0.96, and 0.98, respectively (Fig. 4).

### Cophylogenetic analyses

**Figure 5:**
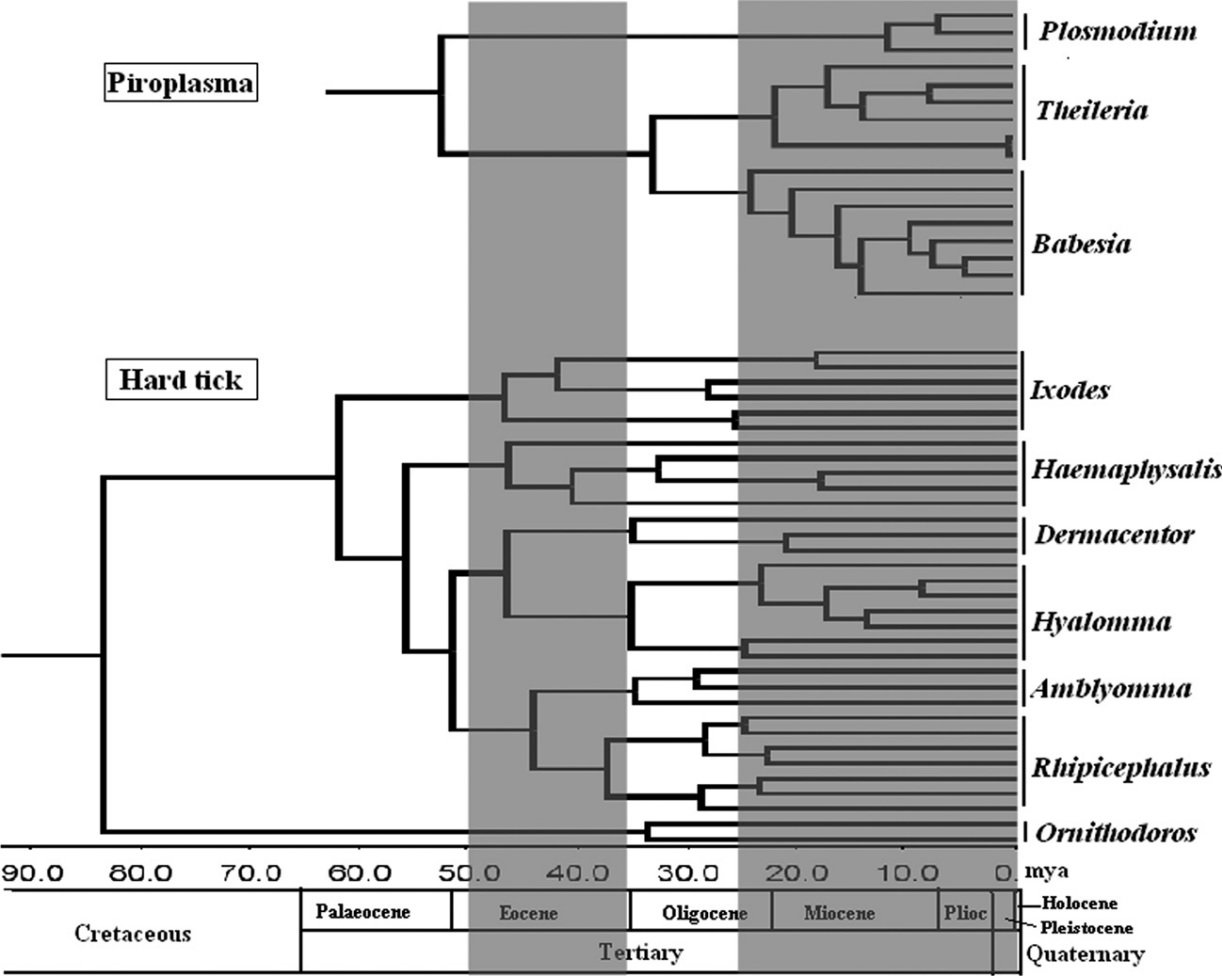
Evolution of piroplasmid parasite lineages and those of their hosts. Gray areas represented the divergence time of genus and species of two organisms.

**Figure 6:**
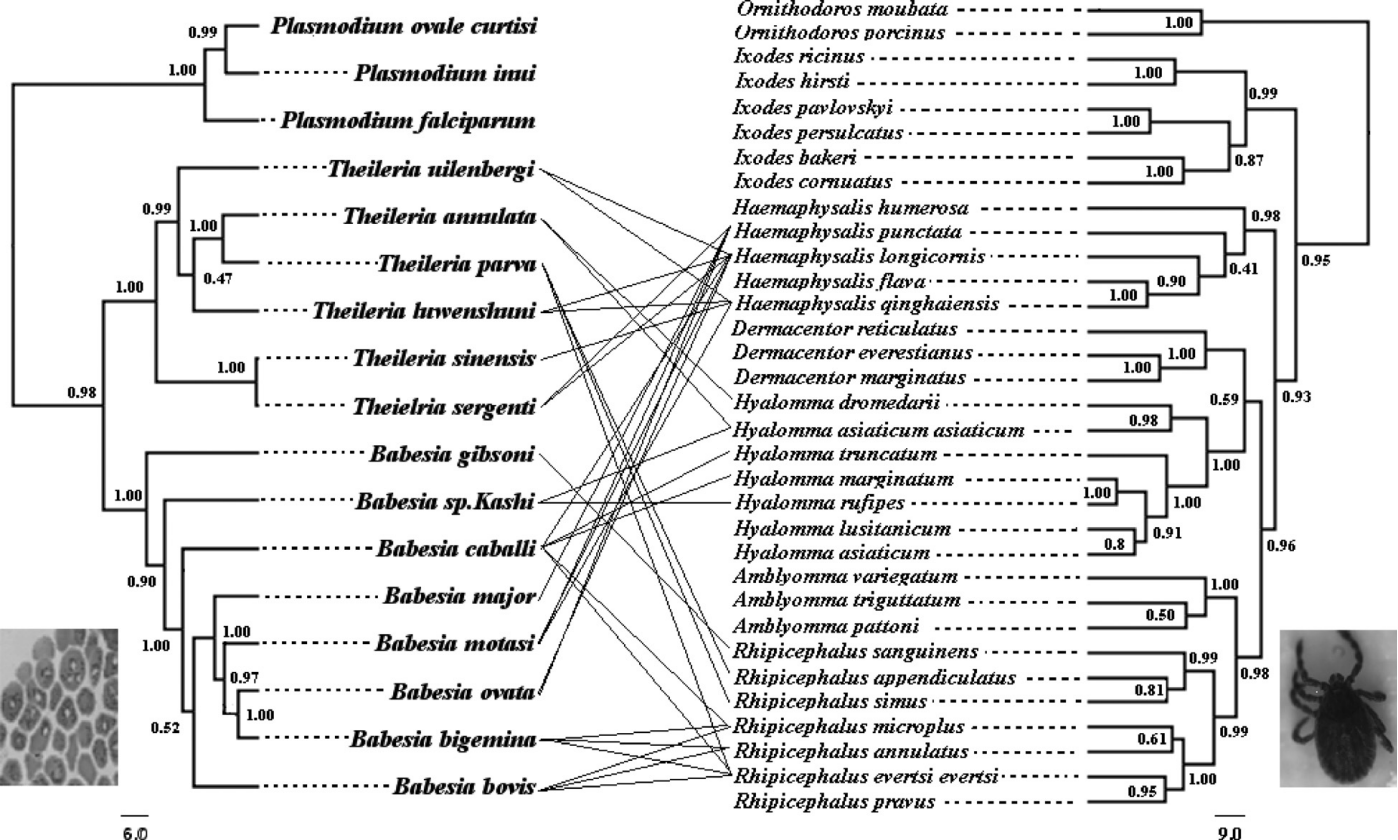
Phylogenies of piroplasmid and their hard tick hosts. Lines indicate specific transmission of hard tick host and piroplasmid.

When the two phylogenetic trees of the ticks and parasites are compared (Fig. 5), we can see that the genus divergence time of the parasite (*Theileria* and *Babesia*) is later than their invertebrate hosts (*Ixodes*, *Haemaphysalis*, *Dermacentor*, *Hyalomma*, *Amblyomma*, and *Rhipicephalus*). Moreover, this trend is also lasting at the species divergence time for majority of parasites and their definitive hosts. Furthermore, the Bayesian topologies of the combined parasite sequence data set and host data set were used in the cophylogenetic analysis and considered as species trees. This revealed that parasite and host phylogenies were not congruent (Fig. 6).

## Discussion

In the study of evolution, estimates of divergence time can provide crucial information to speciation events (Hayakawa et al. 2008; Ricklefs and Outlaw 2010). In previous studies, the divergence time of the Apicomplexa was estimated to be between 1100 and 500 Mya based on molecular clock estimates (Berney and Pawlowski 2006; Escalante and Ayala 1995). Published divergence time of piroplasmid varies from 820 Mya to 17 Mya and is surrounded by a degree of uncertainty. We calculated the divergence time to be 56.48 Mya (95% HPD 28.17–86.87), which is earlier than the result from a study based on 18S rRNA (17.11 Mya) (Lack et al. 2012). This discrepancy might be because of the difference in evolutionary rate of the two genes. The gene encoding the 18S rRNA is more conservative than is *COI*, and has a slower evolutionary rate (Gou et al. 2012; Nassonova et al. 2010). Published divergence time of ticks ranges across two evolutionary epochs, including the Triassic (248–206 Mya) (Balashov 1989, 1994) and Cretaceous (146–65 Mya) (Black and Piesman 1994). However, there are few studies about the divergence time of hard ticks. We estimated the divergence time of hard ticks to be 86 Mya (95% HPD 61.11–104.16) based on three dominica amber samples, which is later than previous estimates (90–120 Mya) (Klompen et al. 2005; Mans et al. 2002). We can see that the both genus and species divergence time of the parasite are later than their hosts hard ticks after compared two phylogenetic trees.

The other objective of this study was to assess the degree of congruence between the parasite phylogeny and that of their definitive hosts. From Figure 6, we can see that parasite and host phylogenies were not congruent. However, although most piroplasmid species in this study (except *B. ovata* and *B. gibsoni*) can be transmitted by two or more species of hard tick (Bautista et al. 2001; Dantas-Torres and Figueredo 2006; Hove et al. 1998; Iori et al. 2010; Mazyad et al. 2010; Schwint et al. 2008), these hard tick species are always from the same genus. This means that hard ticks, as a vector, transmitted piroplasmids with high genus specificity and low species specificity.

Although environmental effects are often excluded from experimental coevolutionary studies and are included in theoretical models as ‘noise’, some studies under laboratory conditions have shown that the environment in which hosts and parasites interact can substantially affect the strength and specificity of selection (Wolinska and King 2009). However, geographical isolation as an element of the environment should also be discussed in this study of coevolution between piroplasmids and hard ticks. For instance, both *B. major* and its vector *H. punctata* are only found in the Xinjiang Uygur Autonomous Region of China. This region is located in the Junggar basin and is isolated from other areas by desert and high mountains. A similar case is also observed with *T. sinensis* and its vector *H. qinghaiensis*. These two organisms are mainly distributed in Gansu and Qinghai provinces in northwest of China (Yin et al. 1996), areas characterized by high altitude, drought, and loess. These two examples of geographical isolation have resulted in piroplasmids and hard ticks forming a single biological system over a long period of evolutionary time.

## Supporting Information

**Table S1.** Species list of piroplasmids and hard tick host *COI* sequences, including GenBank accession numbers and origin.
