# Supplementary material for: Coevolutionary analyses of the relationships between piroplasmids and their hard tick hosts
Source: Ecol Evol. 2013 Jul 30;3(9):2985–93. doi: 10.1002/ece3.685 (PMC3790545; doi:10.1002/ece3.685)
Supplement: Supplementary file 1 [file ece30003-2985-SD1.doc]

**Table S1.**

Species list of piroplasmids and hard tick host *COI* sequences, including GenBank accession numbers and origin

|  | **Taxa** | **Accession** | **Origin** |
| --- | --- | --- | --- |
| Piroplasmid | *Theileria uilenbergi* | JQ518294 | This study |
|  | *Theileria annulata* | JQ518291 | This study |
|  | *Theileria parva* | AB499089 | [Hikosaka et al., 2010](#_ENREF_5) |
|  | *Theileria luwenshuni* | JQ518295 | This study |
|  | *Theileria sinensis* | JQ518293 | This study |
|  | *Theileria sergenti* | JQ518292 | This study |
|  | *Babesia gibsoni* | AB499087 | [Hikosaka et al, 2010](#_ENREF_5) |
|  | *Babesia* sp. Kashi | JQ518308 | This study |
|  | *Babesia caballi* | AB499086 | [Hikosaka et al., 2010](#_ENREF_5) |
|  | *Babesia major* | JQ518302 | This study |
|  | *Babesia motasi* | JQ518310 | This study |
|  | *Babesia ovata* | JQ518306 | This study |
|  | *Babesia bigemina* | JQ518300 | This study |
|  | *Babesia bovis* | JQ518301 | This study |
| Out group | *Plasmodium ovale curtisi* | HQ712052 | [Pacheco et al., 2011](#_ENREF_10) |
|  | *Plasmodium inui* | AB444111 | [Sawai et al., 2010](#_ENREF_12) |
|  | *Plasmodium falciparum* | AJ276846 | [Conway et al., 2000](#_ENREF_2) |
|  | *Ornithodoros porcinus* | AB105451 | [Mitani et al., 2004](#_ENREF_7) |
|  | *Ornithodoros moubata* | AB073679 | [Fukunaga et al., 2001](#_ENREF_4) |
| Hard tick | *Ixodes pavlovskyi* | AB231669 | [Mitani et al., 2007](#_ENREF_6) |
|  | *Ixodes persulcatus* | JQ737101 | This study |
|  | *Ixodes bakeri* | GU437873 | Unpublished |
|  | *Ixodes cornuatus* | FJ571511 | Unpublished |
|  | *Ixodes ricinus* | GU074940 | [Noureddine et al, 2011](#_ENREF_9) |
|  | *Ixodes hirsti* | FJ571510 | Unpublished |
|  | *Haemaphysalis humerosa* | AF132819 | [Murrell et al., 2000](#_ENREF_8) |
|  | *Haemaphysalis longicornis* | JQ737087 | This study |
|  | *Haemaphysalis flava* | JQ737097 | This study |
|  | *Haemaphysalis qinghaiensis* | JQ737089 | This study |
|  | *Haemaphysalis punctata* | FN394340 | [Chitimia et al., 2010](#_ENREF_1) |
|  | *Dermacentor reticulatus* | HM193882 | Unpublished |
|  | *Dermacentor everestianus* | JQ737079 | This study |
|  | *Dermacentor marginatus* | HM193887 | Unpublished |
|  | *Hyalomma truncatum* | AF132824 | [Murrell et al., 2000](#_ENREF_8) |
|  | *Hyalomma marginatum* | AJ437097 | [Rees et al., 2003](#_ENREF_11) |
|  | *Hyalomma rufipes* | JQ737110 | This study |
|  | *Hyalomma lusitanicum* | EU827742 | Unpublished |
|  | *Hyalomma dromedarii* | AJ437062 | [Rees et al., 2003](#_ENREF_11) |
|  | *Hyalomma asiaticum asiaticum* | JQ737106 | This study |

|  | *Hyalomma asiaticum* | JQ737072 | This study |
| --- | --- | --- | --- |
|  | *Amblyomma triguttatum* | AB113317 | Unpublished |
|  | *Amblyomma pattoni* | HM193876 | Unpublished |
|  | *Amblyomma variegatum* | GU062743 | [Figures et al., 2010](#_ENREF_3) |
|  | *Rhipicephalus microplus* | JQ737083 | This study |
|  | *Rhipicephalus annulatus* | AF132825 | [Murrell et al., 2000](#_ENREF_8) |
|  | *Rhipicephalus evertsi evertsi* | AF132835 | [Murrell et al., 2000](#_ENREF_8) |
|  | *Rhipicephalus pravus* | AF132837 | [Murrell et al., 2000](#_ENREF_8) |
|  | *Rhipicephalus appendiculatus* | AF132833 | [Murrell et al., 2000](#_ENREF_8) |
|  | *Rhipicephalus simus* | AF132840 | [Murrell et al., 2000](#_ENREF_8) |
|  | *Rhipicephalus sanguinens* | JQ737084 | This study |
